# Water and the UN sustainable development goals

**DOI:** 10.14324/111.444/ucloe.000029

**Published:** 2022-01-07

**Authors:** Luiza C. Campos, Daniel Olago, Dan Osborn

**Affiliations:** ^1^Department of Civil, Environment & Geomatic Engineering, Faculty of Engineering Science, University College London, Gower Street, London WC1E 6BT, UK; ^2^Department of Geology, University of Nairobi, University of Nairobi, P.O Box 30197, Nairobi, Kenya; ^3^Chair of Human Ecology, Department of Earth Sciences, University College London, 2 Taviton Street, London WC1H 0BT, UK

Water is essential for life, but we need to balance human needs with those of the environment on which we depend for our wellbeing, our health and much of our wealth. Not all of us are lucky enough to have access to adequate water resources and services linked to water, such as readily available low-cost drinking water and sanitation systems.

According to the World Health Organization and United Nations Children’s Fund (WHO/UNICEF), hundreds of millions of people are still without access to safely managed drinking water and sanitation services [1]. Their Joint Monitoring Programme (JMP) report, Progress on Drinking Water, Sanitation and Hygiene 2000–2020 [2], found that although considerable progress has been made in achieving universal access to basic water services, the proportion of improved water sources that are accessible, available and free from contamination varies widely between countries. This indicates that many countries are facing a challenge to meet the Sustainable Development Goal (SDG) target for safely managed services. In addition, despite increasing the rural coverage of safely managed water services in some countries, and in other countries this coverage is similar to the urban coverage, there is a huge gap in terms of water quality.

Many aquatic ecosystems (freshwater, brackish and oceanic) also are under threat with knock-on consequences for humanity. Large quantities of inadequately treated or untreated wastewater are still being discharged into our surface, ground and coastal waters. The WHO reports that at least 2 billion people globally consume water from a source contaminated with faeces. Faecal contamination in the water supply system, whether rudimentary or complex, is a major cause of infectious diseases such as cholera, typhoid fever, diarrhoea, dysentery, hepatitis A and polio [3]. As a result, 1.2 million people die every year from water related diseases [4]. According to the Global Water Institute, in low- and middle-income countries, almost 50% of the population can link health problems to waterborne diseases. In addition, emergent pollutants such as microplastics, antibiotics, per- and polyfluoroalkyl substances (PFAS) and their degradation products found in water sources and in the environment pose a health risk to humans and animals [5].

## Water and the UN SDGs

Many of the SDGs^1^ are water related: as well as SDG 6 (clean water and sanitation), progress on many of the other SDGs depends heavily on water such as food (SDG 2), good health and wellbeing (SDG3), climate (SDG 13), energy (SDG 7), industry (SDG 9), sustainable cities (SDG 11), and responsible consumption and production (SDG 12). Two others (SDGs 14 and 15 concerning life on land and the oceans) depend absolutely on the planet’s hydrological cycles.

There are four major competing needs for water: water for the environment; water for drinking and sanitation; water for farming; water for other businesses and industry^2^. When water is in short supply, as in droughts, all these water sectors suffer. Striking the balance between these competing needs for water is essential if the SDGs, now widely recognised as relevant to all countries, will be achieved within the given time frame.

The Brisbane Declaration and Global Action Agenda on Environmental Flows (2018) calls for urgent action to protect and restore environmental flows and aquatic ecosystems considering they are essential to manage water resources and a foundation for achievement of water-related SDGs [6]. To avoid water becoming the third major environmental crisis (the others being climate and biodiversity, which are also intricately inter-connected with water), we need to know much more about factors that affect each of these major uses and the balance between them at global, regional and local scales. In many ways we need to know how much water is needed by at least three of the four major uses and what constitutes the requirement of the other one, the environment.

Avoiding shortages in supplies of water will require such knowledge to be integrated with policy approaches that include ways of delivering on the SDG ambitions. Thus, we need a better understanding of how the SDG goals and targets interact with policy and practice in areas as diverse as food production, biodiversity conservation, industry, public health and commerce.

Understanding these interactions may lead to new ways of assessing the value of water in social and environmental contexts, as well as economic contexts and may also inspire new technological and community-based solutions to water issues. Such a revised valuation of water, making use of quantitative and qualitative measures, may lead to progress on accounting for water that will help delivery bodies and funders prioritise water policies, programmes and projects such that progress towards achieving the SDGs can accelerate in all countries.

## About this series

This special series on water and the UN SDGs published in *UCL Open: Environment* sets out to examine the progress towards, as well as building upon the literature base of, the SDGs viewed from the perspective of the global water cycle. We invite researchers, practitioners and policy teams to submit multi-disciplinary articles to this series on Water and the UN SDGs. Articles can be scholarly research articles, commentaries on policies or policy programmes, or descriptions of projects and/or their outcomes that are helping deliver the SDGs or change the way we view the planet’s water resources.

## Notes

1 Available from: https://sdgs.un.org/goals [Accessed 19 August 2021].

2 UCL Beyond Boundaries Session 3: Sustainability & the Water cycle – Full session [Video file]. 4 Nov 2020. Available from https://www.youtube.com/watch?v=L_81qoZ6VIU.
